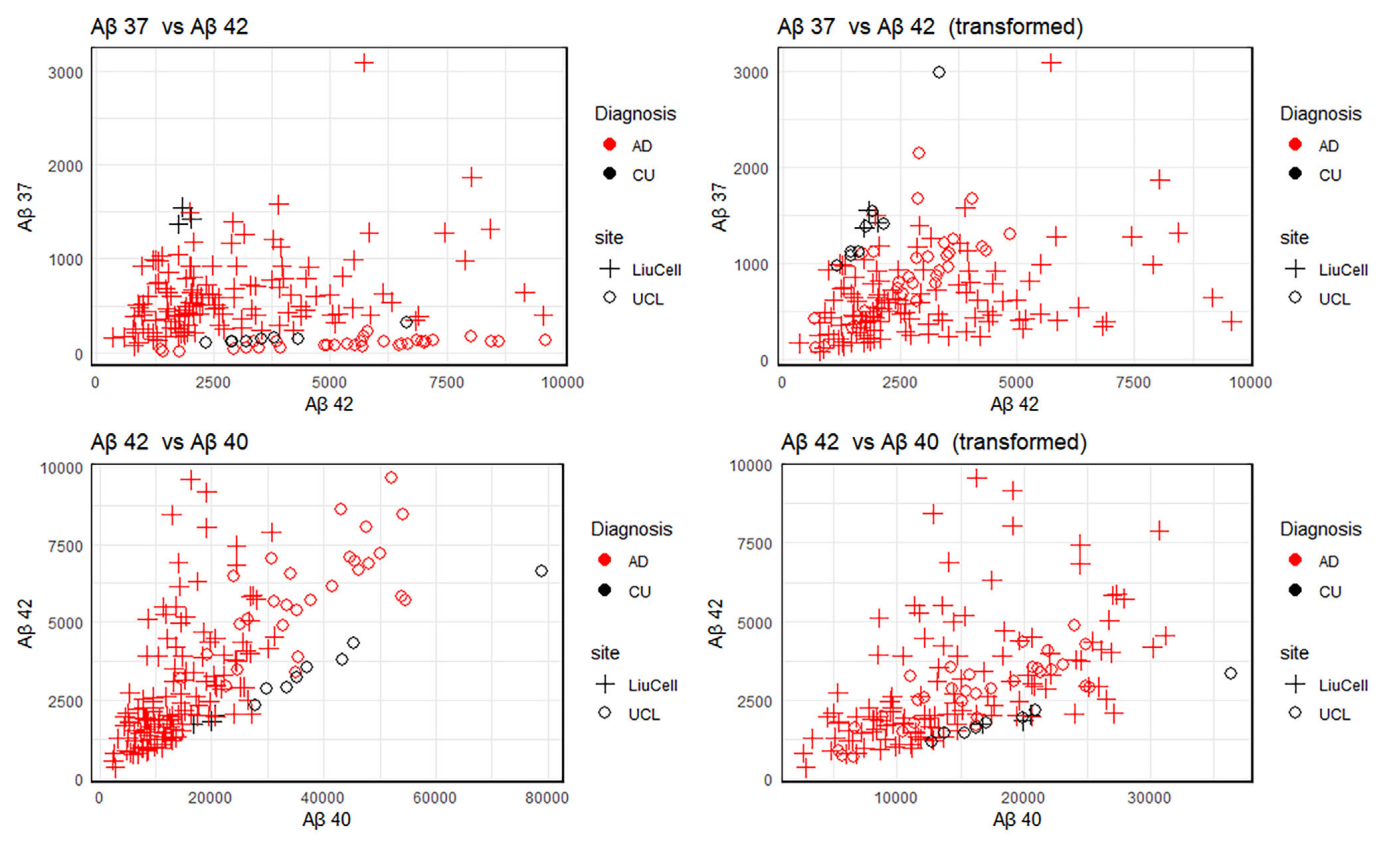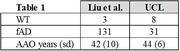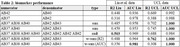# Data‐driven analysis of Aβ peptide ratios in iPSC models of familial Alzheimer’s disease for diagnosis and prediction of symptom onset

**DOI:** 10.1002/alz.093960

**Published:** 2025-01-09

**Authors:** Isaac Llorente Saguer, Rebecca Gabriele, Selina Wray, Neil P Oxtoby, Charlie Arber

**Affiliations:** ^1^ UCL Centre for Medical Image Computing, Department of Computer Science, University College London, London United Kingdom; ^2^ UCL Queen Square Institute of Neurology, London United Kingdom; ^3^ University College London, London United Kingdom

## Abstract

**Background:**

Familial Alzheimer’s disease (fAD), arising from mutations in amyloid‐precursor‐protein (APP) and presenilin (PSEN1/2) genes, leads to the production of longer, aggregation‐prone amyloid‐beta (Aß) peptides—a hallmark of Alzheimer’s disease. Age‐at‐onset (AAO) varies among carriers of different mutations. Recent evidence challenges the Aß42:40 ratio as the leading and predictor of AAO between different pathogenic variants, prompting exploration of peptide combinations as potential biomarkers for these tasks.

**Method:**

Data consisted of patient‐derived in‐vitro iPSC models of fAD and Ab measurements via enzyme‐linked immunosorbent assay (ELISA). With novel data of our own (N = 39) and published data from Liu et al. 2022 (N = 134) (table‐1), we performed a data‐driven analysis of Aß37/38/40/42/43 combinations, employing area under the curve (AUC) and coefficient of determination metrics for classification (WT vs AD) and AAO regression. A linear model optimized for these metrics was also explored. Each peptide was divided by the average of the control group of the same assay. Then, we weighted each peptide in accordance with the average profile of the control population in Liu.

**Result:**

Data transformation using controls proved necessary for to produce comparable ratios (figure‐1). Table‐2 presents diagnostic and predictive performance for each peptide‐ratio biomarker relative to biomarkers from the literature (top 3 rows). Our new data‐driven biomarkers improved upon the literature in both diagnostic AUC (not statistically significant: DeLong’s test p>0.47) and predicting AAO (Spearman’s R^2 significant differences in UCL data; Hittner’s p<0.005; p>0.5 in Liu’s).

**Conclusion:**

Our results do not recapitulate Liu et al. findings, as Aß37:42 did not outperform Aß42:40 in our data; one explanation could be that our lines represent a physiological model of Ab production that is free from plaque deposition. While no significant differences emerged in AD classification, specific peptide ratios demonstrated varying correlations with AAO: short‐to‐long ratio > Aß42:40, and Aß42:40 > Aß37:42, but other ratios were found to perform better in each set, suggesting mutation‐specific profiles. Data‐driven linear combination can improve the results, and notably, both optimizing for AUC or AAO found the same ratio of short/long, albeit with different weights. Standardization through control‐based data was essential for robust measures.